# Effects of Two Community-Based Exercise Programs on Adherence, Cardiometabolic Markers, and Body Composition in Older People with Cardiovascular Risk Factors: A Prospective Observational Cohort Study

**DOI:** 10.3390/jpm10040176

**Published:** 2020-10-16

**Authors:** Esther García-Sánchez, Jacobo Á. Rubio-Arias, Vicente Ávila-Gandía, F. Javier López-Román, Juan F. Menarguez-Puche

**Affiliations:** 1 Fundación para la Formación e Investigación Sanitaria de la Región de Murcia (FFIS), Calle Luis Fontes Pagán 9, 1ª planta, 30003 Murcia, Spain; egsanchez@ucam.edu; 2 LFE Research Group, Department of Health and Human Performance, Faculty of Physical Activity and Sport Science-INEF, Universidad Politécnica de Madrid, 28040 Madrid, Spain; 3 Sports Physiology Department, Catholic University of Murcia (UCAM), 30107 Murcia, Spain; vavila@ucam.edu (V.Á.-G.); jlroman@ucam.edu (F.J.L.-R.); 4 Biomedical Research Institute of Murcia (IMIB-Arrixaca), 30107 Murcia, Spain; 5 Primary Care Health Center, Jesús Marín, Calle Enrique Bernal Capel, 4, Molina de Segura, 30500 Murcia, Spain; juanfran.menarguez@gmail.com

**Keywords:** physical activity, exercise, primary care, METs, chronic disease

## Abstract

Cardiovascular disease is one of the leading causes of death globally, and cardiovascular risk factors (CRFs) are major behavioral risk factors. Therefore, community-based programs are being designed based on the prescription of physical exercise from primary care centers to improve people’s health through changes in lifestyle. The objective was to compare the effects of two types of community exercise on adherence, lipid profile, body composition and blood pressure. A prospective observational cohort study was designed with two cohorts of study depending on the duration and type of physical exercise program performed. Fifty-one participants (82.4% women) with CRF completed the observation period in which they carried out a short-term, non-individualized exercise program (3 months), and 42 participants (71.4% women) with CRF completed the observation period in which they conducted a long-term, individualized exercise program (6 months). The results suggest that participants who carried out the longer program with an individualized progression produced greater adherence to physical exercise and a decrease in diastolic blood pressure. In addition, LDL and insulin levels decreased in both groups. Therefore, our results suggest that a longer duration and individualized evolution of the loads of a community exercise program lead to higher levels of physical activity (PA) and improvements diastolic blood pressure.

## 1. Introduction

Cardiovascular disease (CVD) is one of the main causes of death and disability globally and involves one of the largest public health expenditures [1,2,3,4]. Cardiovascular risk factors (CRFs) are factors associated with CVD and can be biological characteristics, habits, or lifestyles that increase both the risk and probability of death. CRFs are classified into non-modifiable (age, sex, genetic factors, and family history) and modifiable (high blood pressure, smoking, high cholesterol, diabetes mellitus, overweight/obesity, and physical inactivity).

In this regard, dyslipidemia is one of the most prevalent modifiable factors in the adult population and most important for cardiovascular diseases [5], and it is caused by genetic factors and unhealthy lifestyles (e.g., diet and exercise) [6]. Physical exercise can reduce cardiovascular risk by increasing HDL-Col and decreasing the other three components (LDL-Col, VLDL-Col, and triglycerides) while facilitating weight and body fat loss [7]. In recent years, interventions have targeted modifiable risk factors to reduce the number of cardiovascular disease events [8,9,10,11]. According to the WHO, over 80% of premature deaths related to cardiovascular disease could be avoided if modifiable risk factors were minimized or prevented by healthy lifestyle habits, including a healthy diet, physical exercise, and smoking cessation [12]. In this way, it is recommended to reduce sedentary behaviors and increase levels of physical activity (PA) to prevent risk factors associated with cardiovascular diseases [13] and reduce the sedentary lifestyle that has been increasing in recent years [14]. Moreover, physical inactivity (PI) is the fourth most important risk factor for mortality worldwide and leads to 6% of deaths worldwide, above overweight and obesity (5% of global mortality). PI rates are only exceeded by hypertension (13%), cigarette consumption (9%), and excess blood glucose (hyperglycemia) (6%) [15].

Strategies applied by public health have been directed towards the implementation and development of programs focused on the elimination of sedentary behaviors, an increase in physical activity, and the inclusion of exercise as a lifestyle for the population in order to achieve improvements in health and reduce cardiovascular diseases [16]. Exercise is effective in reducing blood pressure [17], and increased adherence to physical exercise leads to improvement in healthy lifestyles [18]. Therefore, exercise and physical activity are considered protective factors for CVD (Ozemek C, 2018). In this regard, community exercise is becoming one of the most prominent strategies [19], improving the interrelationship of all elements that can affect behaviors related to physical activity or exercise such as personal factors (biological and psychological characteristics), social factors (family, affiliation group, and work factors), environmental factors (context for PA performance), and political factors [20]. Reducing physical inactivity in the population can, therefore, be a key public health intervention and could potentially reduce health costs [21,22].

Community-based programs that include simple exercises to increase motivation and enjoyment have been designed to enhance autonomy [23], and they could improve exercise adherence and lifestyle changes by reducing cardiovascular risk factors [24]. In addition, training should be individualized and adapted to the characteristics of the participant [25,26]. All this could reduce the risk factors for people at risk of CVD and lead to a lifestyle change away from disease [27]. However, the effects of community-based exercise programs on people with cardiovascular risk factors are unclear. Therefore, the objective of this study was to analyze the effect of two community-based exercise programs with different protocols and duration on adherence, body composition, and metabolic profile in participants with cardiovascular risk.

## 2. Materials and Methods

### 2.1. Study Design

**Figure 1:**
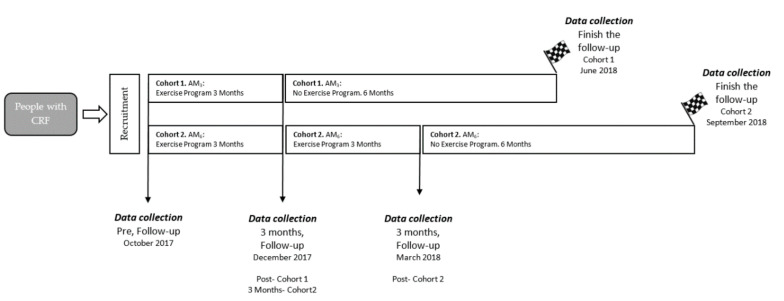
Prospective observational cohort design and testing procedures. CRF: cardiovascular risk factors, AM: Activa Murcia.

A prospective observational cohort study was conducted to analyze the effect of two training programs in men and women with CRF. Follow up was conducted on two cohorts that performed two different community-based exercise programs. This study included participants enrolled between 1 October 2017 and 10 September 2018 (Figure 1). All participants signed a consent form before beginning this study, and this study was approved (CE101702) by the University’s Institutional Science Ethics Committee and agreed to the Declaration of Helsinki.

### 2.2. Participants Selection

Men and women (30–65 years old) were recruited from primary care centers in four cities in the Region of Murcia (Spain). The doctors and nurses of these centers prescribed exercise to a total of 108 participants with CRF who were invited to participate through the “Programa Activa Murcia”. The “Activia Murcia” program is based on the prescription of physical exercise from primary care health centers to participants with CRF. The main objective of the program is to promote healthy behaviors and lifestyles through PE via collaboration between primary care physicians and nurses, community councils (assignment of sports facility), and the Ministry of Health (organizing and coordinating body). Key features of the program include (a) government support, (b) free participation, (c) a link between primary health care professionals (general practitioner and nurse), (d) access was restricted to participants for whom PE was recommended by primary care physicians or nurses, (e) exercises tailored to individual characteristics of participants, and (f) a sport facility close to the participant’s home. Physicians and nurses of primary care centers throughout the Region of Murcia were specially trained in the characteristics of PE and the referral protocol for the program. Participants who were considered eligible by primary care professionals were invited to participate in the program, and for those who accepted and signed the written informed consent, an electronic PE recommendation form was sent by fax to the corresponding city council. Each city council contacted participants by telephone to start physical activities in a municipal sports center close to the participant’s home [28].

Participants were included if they (1) had one or more of the following CRFs: (a) hypertension (diagnosed according to the criteria of ESH/ESC Practice Guidelines for the Management of Arterial Hypertension) [29], (b) lipid alterations (diagnosed according to the criteria of European Society of Cardiology (ESC) and European Atherosclerosis Society (EAS)) [30], (c) smoking (person who has smoked at least one cigarette a day in the last 6 months), (d) overweight/obesity (diagnosed according to the criteria of AHA/ACC/TOS guideline for the management of overweight and obesity in adults ) [31], and/or (e) prediabetes (basal glucose: 00–125 mg/dL and/or HbA1_c_: 5.7–6.4%) [32]; (2) did not exercise (two or more times a week). Participants with the following criteria were excluded: (1) participants with cardiovascular diseases; (2) participants with chronic or terminal diseases; (3) clinically significant acute infections; (4) inability to understand informed consent; (5) ischemic and/or cerebrovascular heart disease diagnosis; (6) serious mental illness including psychosis, severe depressive disorder, and/or neurosis; (7) limiting musculoskeletal system pathology or musculoskeletal diseases that worsen with exercise; (8) HBP with SBP > 180 and DBP > 110; (9) uncontrolled metabolic disease (diabetes, thyroid diseases); (10) pregnant or lactating women; (11) not wishing to consent to the storage and transmission of medical data; (12) participants whose condition did not make them eligible for study according to the researcher; (13) participants who previously completed the program; (14) difficulty to contact.

### 2.3. Definition of Cohorts

In this observational study, two different cohorts of people with CRF were followed for 12 months after enrollment to compare the effects of two different community-based exercise programs. The training programs were supervised and conducted by exercise experts.

Cohort 1. ‘Activa Murcia’ (AM_3_: 3 months). AM_3_ began in October 2017 and was completed in December 2017. Data collection was performed before after and 6 months into the observation period (June 2018). The total duration of the exercise program was 3 months, with a frequency of 3 sessions per week and a total of 30 sessions and 6 months follow up without physical activity. Each session lasted 1 h, and the rest period between each training day ranged between 24 and 48 h. The training load was progressive and not individualized (Table 1). First, there was a general warm up that involved 5 min of active and ballistic stretching. During the main part of the training sessions, strength exercises and aerobic circuitry (RACT) were included in which participants performed self-loading or low external load exercises. For the last 5 min, participants conducted static exercises. The training load was progressive and not individualized. Additionally, load and load progression during sessions was monitored with HR and the rated perceived exertion (RPE) scale. During the cool down, participants carried out a set of stretching exercises.

Cohort 2. ‘Activa Murcia’ (AM_6_: 6 months). AM_6_ began in October 2017 and was completed in March 2018. Data collection was performed before and after 6 months into the observation period (September 2018). Participants completed a 6 month training program with a frequency of 3 sessions per week and a total of 72 sessions and 6 months follow up without physical activity. Each session lasted 1 h, and the rest period between each training day was 24 to 48 h. Prior to each training session, participants performed a warm up in which they carried out mobility exercises and static and ballistic stretching. For the last 5 min, participants conducted static exercises. The main part of the training session involved strength circuits or aerobic exercises with self-loading, and in none of the sessions did participants train strength and aerobic capacity simultaneously. These circuits contained exercises that were easy to execute (e.g., half squat, steps, and lounge). Circuit training included dynamic actions using large muscle groups.

The increase in training load was progressive, and the training load increased each week by changing the speed of execution and rest between circuit exercises. In order to individualize the intensity of the training load, the participants were divided into groups according to their level of physical fitness. Subjective perception of the effort of each participant was used to control the training load. When the last sessions of the week were completed with an RPE of 11 or less, their load was increased [19]. The characteristics of the training program are presented in Table 2.

### 2.4. Outcome Measures

Participants filled out a questionnaire after the follow-up period to verify that they did not change their lifestyles (exercise and diet).

#### 2.4.1. Exercise Adherence

Exercise adherence was measured by the levels of physical activity of the participants after the community-based exercise programs. The International Physical Activity Questionnaire—Short Form—(IPAQ-S) for adults was used to analyze the levels of physical activity (PA) [33,34,35]. Six months after the end of the exercise program, participants were asked to fill in the IPA questionnaire by telephone. Metabolic equivalents (METs) per week were calculated following the guidelines for data processing and analysis of the IPAQ [36,37]:

MET-minutes/week = walking (3’3 MET × minutes × days per week) + moderate PA (4 MET × minutes × days per week) + high PA (8 MET × minutes × days per week).

#### 2.4.2. Body Composition

Height (cm) and weight (kg) were assessed with a digital stadiometer Seca 700 (Seca^®^ Ltd., Germany). Then, the mass (kg) was divided by the squared height (m^2^) to calculate the BMI. Furthermore, total fat-free mass (kg) and total lean body mass (kg) were measured via Bioelectrical Impedance Analysis with Tanita BC-601 (TanitaCorp., Tokio, Japan) according to the manufacturer’s guidelines.

#### 2.4.3. Blood Pressure

Systolic blood pressure (SBP in mmHg) and diastolic blood pressure (DBP in mmHg) were measured using a mercury sphygmomanometer (Minimus II, Riester, Jungingen, Germany) following the guidelines of the Spanish Society of Cardiology [38].

#### 2.4.4. Lipid Profile and Dyslipidemia

The blood was drawn from the median cubital vein of the right arm while the participant was seated, and the participant fasted prior (12 h). The blood was analyzed in the laboratory using an automated Hematology Analyzer, Pentra 80—HORIBA ABX (Horiba Medical, Northampton, UK). The blood test provided total cholesterol (mg/dL), LDH (mg/dL), HDL (mg/dL) and triglycerides (mg/dL), basal glycemia (mg/dL), glycated hemoglobin (HbA1_c_ in %), and basal insulinemia (mg/dL).

### 2.5. Data Analysis

Statistical analysis was performed with the Statistical Package for the Social Sciences (SPSS, version 21, SPSS Inc., Chicago, IL, USA). Prior to data analysis, the descriptive data were shown as the mean ± standard deviation (continuous variables). In addition, the Shapiro–Wilk test was used to determine the normal distribution of the variables. Mauchly’s W test analyzed the sphericity between measurements. For inferential analysis, t-tests or non-parametric equivalents (U Mann–Whitney test) were run to analyze the effects. In addition, analysis of covariance (ANCOVA) with baseline values included as co-variables was used to compare groups at different moments of evaluation during the follow-up period. The level of significance was set to *p* ≤ 0.05.

## 3. Results

**Figure 2:**
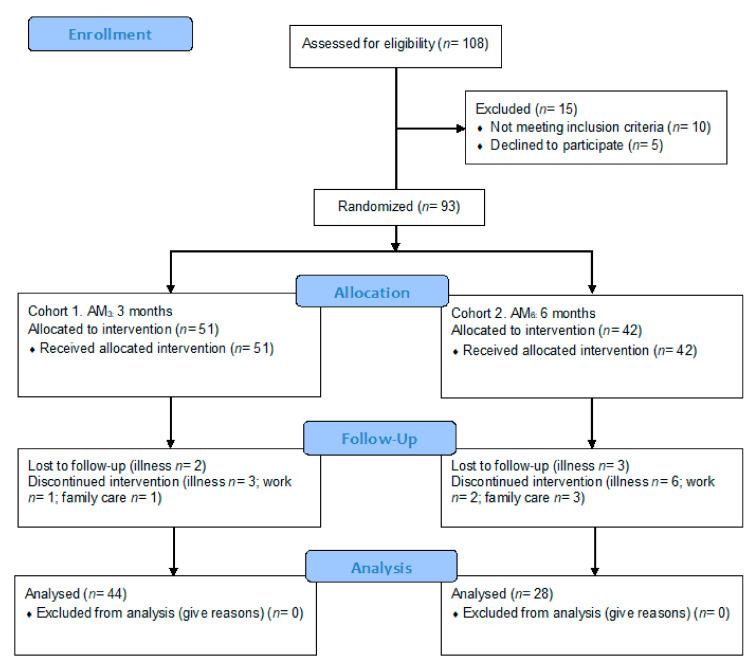
Flow diagram of the progress of the prospective observational cohort study.

Figure 2 shows the participant flow during the community-based exercise program. After enrolment, 7 participants dropped out of this study in the AM3 program and 14 in the AM6 program—the main reasons being illness, work, and family care. 

In the end, 51 participants with CRF completed the 3 month (AM3) exercise program and 42 participants with CRF finished the 6 month (AM6) exercise program (Table 3). There were no statistically significant differences observed between cohorts in sex, weight, BMI, or age at baseline. In addition, the participants informed that they did not and had not consumed nutraceuticals or nutritional supplements before or during the follow-up period. Furthermore, the participants did not report changes in their drug treatment or diet during this study.

### 3.1. Adherence: Physical Activity

**Figure 3:**
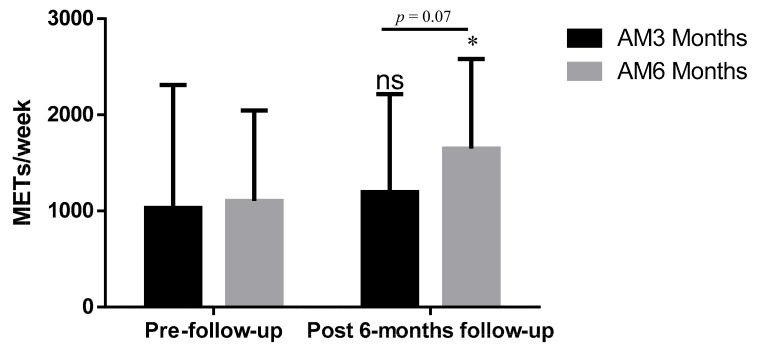
METs/min per week performed in AM3 and AM6 at the beginning and 6 months after the exercise program. ns: no significant effects between pre- and post-6 months follow up; * *p* < 0.05 significant differences between baseline and 6 months follow up.

The ANOVA analysis showed interaction effects for the time factor (pre- vs. post-6 months follow up: F = 12.22; *p* = 0.001) and time × group difference (F = 4.13; *p* = 0.046) in METs (min/week). The pair-wise comparison showed a significant increase in METs in AM_6_ (Δ = 453 METs/week; *p* < 0.001) between pre- and post-6 months follow up (Figure 3). However, no significant differences were found between groups at 3 (mean difference, MD: 73.72, *p* = 0.800) and 6 (MD: 453.00, *p* = 0.071) months.

### 3.2. Body Composition

ANCOVA revealed no significant effects in any variable. Table 4 provides the summary statistics for AM_3_ and AM_6_.

### 3.3. Systolic and Diastolic Blood Pressure

Table 5 shows the summary statistics for SBP and DBP. No main effects were observed in systolic blood pressure. However, a main effect (time) on DBP was observed. The pair-wise comparison showed a significant decrease in DBP in the AM_6_ group after 3 months (MD: 2.42, *p* = 0.896) and 6 months (MD: 7.9 mmHg, *p* = 0.004). Nevertheless, no significant differences were found between groups.

### 3.4. Dyslipidemia Lipid Profile

Regarding the lipid panel and the glycemic profile, significant main time effects were observed for LDL, HDL, and insulin. Furthermore, a time*group effect was found for triglycerides (Table 6). LDL decreased significantly in the AM_6_ group at 3 (MD: 14.34, *p* = 0.019) and 6 (MD: 23.01, *p* < 0.001) months, and there were differences between groups at 6 months follow up (MD: 11.52; *p* = 0.040). Moreover, differences between groups were observed at all time points for HDL (baseline, MD: 8.35, *p* = 0.017; 3 months, MD: 9.85, *p* = 0.007; 6 months, MD: 8.81, *p* = 0.021). In addition, triglycerides tended to decrease in the AM_3_ group (MD: 17.72, *p* = 0.057), and differences between groups were shown at 3 months (MD: 36.87, *p* = 0.038) and 6 months (MD: 35.25, *p* = 0.024).

Concerning the glycemic profile, only a significant main effect on insulin (time effects, F: 10.40, *p* = 0.0001) was observed. Blood insulin concentrations decreased in the AM_3_ group at 3 months (MD: 2.156; *p* = 0.002) and in the AM_6_ group at 3 months (MD = 1.788; *p* = 0.049), and a significant difference between groups was observed after 3 months in favor of AM_3_ (MD: 1.638; *p* = 0.047).

## 4. Discussion

This study was conducted to determine the effects of two community exercise programs on adherence (level of physical activity), cardiometabolic markers, and body composition in older people with cardiovascular risk factors. The main findings indicated that progressive, individualized, long-term exercise improved the amount of physical activity performed by participants six months after the intervention was completed. In addition, LDL and insulin levels decreased in both groups.

The current study found that the amount of physical activity 6 months after AM_6_ (1649 METs) was higher than that of AM_3_ (1031 METs). Therefore, the AM_6_ group decreased their level of physical inactivity, which could lead to a change in lifestyle. A possible explanation for these results may be due to the long duration of the intervention. According to the transtheoretical model of health behavior change, a minimum of 6 months is recommended to achieve adherence to exercise [39,40].

Another possible explanation could be due to the differences in the characteristics of the physical exercise programs. Individualized and progressively loaded training programs could increase feelings of competence and enjoyment as well as increase motivation [23,25,41], and the AM_6_ program could have benefited from combining these features. In contrast, the AM_3_ program with high-intensity and non-individualized training may have led to more negative affective responses, resulting in a lack of long-term adherence to exercise [24,26]. In this way, the improvements in health and feelings of wellness that exercise can provide are also needed to increase exercise adherence. For this reason, the training load must be properly adjusted and individualized for each participant to maximize health gains [42,43].

Contrary to expectations, this study did not find a significant difference in body composition after follow up. The lack of significant results could be due to the intensity of the training programs. Sultana et al., 2019 [44] suggest that low-volume HIIT is inefficient at lowering total body fat mass or percentage of total body fat compared to a control without exercise and with continuous exercise of moderate intensity. Therefore, the results observed suggest that the intensity and load of training were not enough to modify the body composition of the participants. As a result, the recommendations for exercise to combat obesity suggest higher volumes (equivalent to ≥ 1000 MET-min/week) [45] of exercise for significant weight loss [46]. However, several studies have observed improvements in body composition with high-intensity and intervallic exercise. Nevertheless, training programs should incorporate a sufficient length of time to lead to a significant decrease in body fat [47]. Thus, the focus of treatment should be on producing high metabolic stress rather than an energetic imbalance for adults who are overfat [48].

After the two types of programs were completed, there was a non-significant decrease in blood pressure (AM_6_ = −3.1 mmHg, AM_3_ = 3.8 mmHg). However, DBP showed a statistically significant decrease of 8 mmHg in the AM_6_ group and a non-significant decrease in the AM_3_ group. These results are not consistent with previous studies showing that exercise leads to a decrease in systolic and diastolic blood pressure [49,50,51,52]. Oliver et al. [17] performed a meta-analysis and observed that moderate-strength training (60–80% 1RM) with a frequency of 2 or 3 weeks and a length of at least 10 weeks is effective in reducing systolic and diastolic blood pressure. In addition, several studies have also found that high-intensity interval training, moderate-intensity continuous training, and aerobic training reduce blood pressure [49,50,51,52]. However, Cornelissen et al. [53] observed no change in blood pressure after a 10 week program at a frequency of three sessions per week, 1 h each session, and with an intensity of 33% or 66% of HRR. Therefore, the lack of significant results could be due to the duration of the training programs, with a minimum of 10 weeks at a moderate intensity being necessary to find significant changes [17]. However, the duration of the program could be shorter when working at higher intensities [54].

Nevertheless, the training protocols carried out in the Activa Murcia program are designed to change lifestyles and increase the population’s adherence to exercise. Hence, the intensity of AM6 and the duration of AM3 do not seem to be adequate enough to achieve the desired BP changes. Another possible explanation for the lack of significant results could be due to the heterogeneity of the sample and the initial blood pressure values. Several studies have shown decreases in blood pressure in people with hypertension [49,50,52,55]. In these studies, it is easier to observe improvements in blood pressure because all the people included were hypertensive. People with different CRFs were included in our study; therefore, it is more difficult to observe improvements in blood pressure because all the participants were not hypertensive.

In relation to lipid profile, our results suggest that they significantly decreased LDL and insulin resistance. This improvement was greater in the AM_6_ program than in the AM_3_ program for LDL. According to the European Society of Cardiology and European Society of Atherosclerosis, LDL is the most important health marker of the lipid profile [56,57]. However, no other changes were observed in any other marker of the lipid profile. In this regard, several studies show that a progressive increase in exercise intensity and the practice of regular physical activity are key factors in improving the lipid profile [7,58,59]. Mann et al. confirmed the effects of regular physical activity on cholesterol levels in their review. In addition, individualization of exercise as well as the duration of the physical exercise program could be key factors in improving physical activity levels [7,59]. Motalebi et al. [60] analyzed 24 weeks of walking for 5 days a week and saw significant cholesterol improvements compared the control group in older women. Therefore, the differences observed between programs could be due to differences in the follow-up program (3 months vs. 6 months).

Diet is essential in controlling and reducing cardiovascular risk factors [61] Furthermore, previous studies have shown that diet combined with exercise could be an effective method of preventing and improving cardiovascular risk factors [62], enhancements in body composition, as well as the glycemic profile [7,63]. Nevertheless, the diet in our study was not altered during the follow-up period, therefore, we assumed that diet did not interfere with the effects of exercise. Thus, future studies should prescribe community-based physical exercise programs and individualize diet to the characteristics of the participants.

However, although the exercise was adapted for each participant, the lack of significant results regarding adherence could be due to the absence of more individualized treatment during the study progression by health staff in the health centers. Future studies should follow the recommendations proposed by Ciccone et al. [64], who have suggested individualized care between caregivers and patients, recommending lifestyle changes, monitoring the evolution of the conditions and providing them with the information and advice needed to promote patient empowerment, improve self-management skills and achieve better compliance with care recommendations.

The main strengths of the present study were that the participants were prescribed physical exercise from a public health center and that exercise was considered the means to decrease cardiovascular risk factors. On the other hand, two exercise programs of different duration and type of exercise were compared with the aim of optimising the results for future studies. However, there are several limitations to be considered. This study was an observational cohort study and no modifications and/or adaptations of previously planned exercise programs could be made. Nevertheless, family history was considered when participants were prescribed the exercise intervention but not for statistical analysis. However, there was no follow-up communication between medical staff, sport professionals and patients. In addition, there was a low number of participants included in each observation group, and there was high inter- and intra-cohort heterogeneity.

## 5. Conclusions

Our results suggest that a 6 month program with an individualized training load leads to higher levels of physical activity (adherence) and also decreases diastolic blood pressure. In addition, both training programs resulted in decreased LDL and insulin levels. Therefore, community-based exercise programs improve health and could be a good public health strategy for changing lifestyles by recommending long-term exercise programs with individualized training loads.

## Figures and Tables

**Table 1:** Training progression for Active Murcia 3 months.

*Block*	*Objective*	*Intensity*		*Duration (min)*
		%MHR	RPE	
*Warm up*	Mobility and stretching			5
*Aerobic 1 (intervalic)*	CV endurance	85%	RPE (C): 14–16 RPE (C-R): 5–6	25
*Aerobic 2 (constant)*	CV endurance	80%	RPE (C): 14–16 RPE (C-R): 5–6	25
*Tone up muscle*	muscle strength	90%	RPE (C): 14–16 RPE (C-R): 5–6	25
*Strength and endurance*	muscle strength and CV endurance	60%	RPE (C): 11–13 RPE (C-R): 3–4	25
*Cool down*	flexibility			5

CV: cardiovascular; MHR: maximum heart rate; RPE: rate of perceived exertion; C: category; C-R: category ratio; min: minute.

**Table 2:** Training progression for Activa Murcia 6 months.

Level	Training Mode		Density		Week 1	Week 2	Week 3	Week 4
Level 1	S	W	1	I	L	L	L	M
		R	1	RT	passive	active-passive	active	run-passive
	A	W	3	I	L	L	L	L
		R	1	RT	active-passive	passive	run-passive	run-active
Level 2	S	W	1.05	I	L	L	L	M
		R	55	RT	passive	active-passive	active	run-passive
	A	W	3.10	I	L	L	L	L
		R	50	RT	active-passive	active	run-passive	run-active
Level 3	S	W	1.10	I	L	M	M	M
		R	50	RT	passive	active	run-passive	run-active
	A	W	3.20	I	L	L	L	M
		R	40	RT	active	run-passive	run-active	run-active
Level 4	S	W	1.15	I	L	M	M	M
		R	45	RT	passive	active	run-passive	run-active
	A	W	3.30	I	L	L	M	M
		R	30	RT	active	run-passive	run-passive	run-active
Level 5	S	W	1.20	I	L	M	M	H
		R	40	RT	active	run-passive	run-active	run-active
	A	W	3.40	I	L	L	M	H
		R	20	RT	active	run-active	run-active	run-active
Level 6	S	W	1.25	I	L	M	M	H
		R	35	RT	active	run-passive	run-active	run-active
	A	W	3.50	I	L	L	M	H
		R	10	RT	active	run-active	run-active	run-active

Density is the relationship between working time and rest time. S: strength; A: aerobic; W: work; R: rest; I: intensity; RT: rest type; L: low—slow-speed exercise repetition; M: medium—moderate-speed exercise repetition; H: high—high-speed exercise repetition; passive: motionless, effortless; active-passive: half the time walking and half motionless; active: walking; run-passive: half the time running and half motionless; run-active: half the time running and half walking; run: running.

**Table 3:** Participants characteristics and backgrounds.

	AM_3_ (*n* = 51)	AM_6_ (*n* = 42)
Age (years)	59.2 ± 7.4	59.4 ± 8.9
SEX (% women)	82.4	71.4
Weight (kg)	32.3 ± 9.9	30.1 ± 10.3
BMI (kg/m^2^)	30.6 ± 5.3	29.5 ± 4.3
CVRF (%)		
HBP	62.7	66.7
Dyslipidemia	78.4	76.2
Obesity	51.0	52.4
Overweight	49.0	47.6
Prediabetes	39.2	35.7
Smoking	9.8	7.1
Drugs (%)		
HBP	58.8	61.9
Dyslipidemia	39.2	42.9

Data are the mean ± standard deviation. AM: Activa Murcia; BMI: body mass index; HBP: high blood pressure.

**Table 4:** Body composition variables before the exercise program and after follow up.

					Time		Time × Cohort Effect	
	Group	Pre	Post-3	Post-6	F	*p*	F	*p*
Weight	AM_3_	78.7 ± 16.0	78.9 ± 15.9		1.557	0.215	1.028	0.336
(kg)	AM_6_	75.9 ± 13.5	75.8 ± 13.6	76.8 ± 13.6				
BMI	AM_3_	30.4 ± 5.3	30.4 ± 5.3		0.706	0.437	0.556	0.500
(kg/m2)	AM_6_	29.5 ± 4.5	29.5 ± 4.2	29.8 ± 4.0				
LBM	AM_3_	44.8 ± 8.0	44.3 ± 7.5		2.816	0.089	0.649	0.437
(kg)	AM_6_	44.7 ± 9.5	44.2 ± 9.2	44.6 ± 9.4				

Data are the mean ± standard deviation. BMI: body mass index; F: fat free mass; LBM: lean body mass.

**Table 5:** Systolic and Diastolic blood pressure before the exercise program and after follow up.

					Time		Time × Cohort Effect	
	Group	Pre	Post-3	Post-6	F	*p*	F	*p*
SBP	AM_3_	140.9 ± 20.7	137.1 ± 16.4		2.353	0.115	0.281	0.692
	AM_6_	137.5 ± 18.9	136.1 ± 15.6	134.4 ± 21.3				
DBP	AM_3_	86.1 ± 11.5	83.4 ± 9.5		0.857	0.002	2.978	0.077
	AM_6_	89.1 ± 14.8	86.7 ± 8.6	81.2 ± 10.3 *				

Data are the mean ± standard deviation. SBP: systolic blood pressure; DBP: diastolic blood pressure; AM_3_: Activa Murcia 3 months; AM_6_: activa Murcia 6 months; * *p* < 0.05 significant differences between baseline and 6 months follow up.

**Table 6:** Dyslipidemia and lipid profile before the exercise program and after follow up.

					Time		Time × Cohort Effect	
	Group	Pre	Post-3	Post-6	F	*p*	F	*p*
Lipid panel								
Total cholesterol	AM_3_	210.2 ± 49.6	199.6 ± 38.1		1.342	0.259	2.101	0.146
(mg/dL)	AM_6_	196.5 ± 44.6	195.8 ± 38.2	199.8 ± 37.8				
LDL	AM_3_	124.5 ± 44.7	115.4 ± 33.4		17.395	<0.001	3.084	0.079
(mg/dL)	AM_6_	130.2 ± 32.0	115.9 ± 31.1 *	107.2 ± 27.8 **,#				
HDL	AM_3_	60.6 ± 13.3	62.7 ± 14.3		3.419	0.051	0.552	0.527
(mg/dL)	AM_6_	52.3 ± 12.0	52.9 ± 12.1	53.9 ± 13.7				
Triglycerides	AM_3_	125.5 ± 60.2	107.7 ± 50.2		0.348	0.704	4.152	0.019
(mg/dL)	AM_6_	133.9 ± 58.6	144.6 ± 84.6	143.0 ± 67.4				
Glycemic profile								
Glucose	AM_3_	102.5 ± 20.6	105.3 ± 20.0		1.506	0.229	0.203	0.737
(mmol/L)	AM_6_	107.7 ± 23.2	108.9 ± 26.2	109.0 ± 27.1				
Glycated hemoglobin	AM_3_	5.53 ± 0.49	5.62 ± 0.66		2.723	0.086	1.076	0.330
(%)	AM_6_	5.78 ± 0.65	5.89 ± 0.73	5.77 ± 0.68				
Insulin	AM_3_	9.83 ± 6.43	7.67 ± 3.80 *		10.401	0.001	0.98	0.350
(mU/L)	AM_6_	10.63 ± 5.80	8.84 ± 4.41 *	9.71 ± 4.98				

Data are the mean ± standard deviation. AM_3_: Activa Murcia 3 months; AM_6_: Activa Murcia 6 months; * *p <* 0.05 and ** *p* < 0.01 significant differences between baseline and 6 months follow up, # *p* < 0.01.
